# Effect of Multichannel Convolutional Neural Network-Based Model on the Repair and Aesthetic Effect of Eye Plastic Surgery Patients

**DOI:** 10.1155/2022/5315146

**Published:** 2022-09-01

**Authors:** YixinQu, BingyingLin, ShuilingLi, XianchaiLin, ZhenMao, XingyiLi, RongxinChen, DanpingHuang

**Affiliations:** ^1^Zhongshan Ophthalmic Center, Sun Yat-Sen University, Department of Oculoplastic, Guangzhou, Guangdong 510060, China; ^2^State Key Laboratory of Ophthalmology, Zhongshan Ophthalmic Center, Sun Yat-Sen University, Guangzhou, Guangdong 510060, China

## Abstract

**Objective:**

This study is aimed at exploring the impact of eye model based on multichannel convolutional neural network (CNN) on eye plastic surgery and aesthetic effect, thus formulating methods to improve the effect of eye plastic surgery.

**Methods:**

A total of 64 patients who underwent pouch plastic surgery from January 2020 to March 2021 were selected as the research objects and were divided into observation group and control group by random number table method. The subjects in the observation group were evaluated by multichannel CNN-based eye model and doctors' experience, while those in the control group were evaluated by doctors' experience only, with 32 cases in both groups. Blepharoplasty, lower eyelid skin wrinkles, skin luster, and aesthetic scores were compared between the two groups.

**Results:**

The similarity between the multichannel CNN model detected shape and the actual eye shape (98.78%) was considerably higher than that of the CNN model detected shape (78.65%) (*P* < 0.05). After treatment, the indexes of pouch degree, lower eyelid skin wrinkle, eyelid lacrimal sulcus, skin gloss, and aesthetic score in the observation group were better than those in the control group (*P* < 0.05). The incidence of complications in the observation group (13%) was considerably lower than that in the control group (28%) (*P* < 0.05).

**Conclusion:**

The eye model based on the multichannel CNN model was helpful to improve the surgical repair and aesthetic effect of patients and can improve the occurrence of postoperative complications.

## 1. Introduction

With the gradual improvement of people's living standards, people's focus has gradually shifted from the original problem of food and clothing to the external image, so that cosmetic surgery has become a demand of people nowadays, especially when there are external defects in the human body [1]. Eyes, as a window to convey people's feelings, have a great influence on the overall image of the individual. Having an appropriate eye shape is very meaningful to the personal appearance and temperament. At present, eye plastic surgery is very common in medical cosmetic surgery, including double eyelid plastic surgery [2], canthus opening surgery [3], ptosis correction [4], and pouch surgery [5]. Among them, pouch surgery, as one of the surgeries for eye rejuvenation, is very common in the cosmetic industry, and the surgical method is based on the principle of fat removal and skin removal [6]. The formation of bags under the eyes is related to the skin of the eyelid and the connective tissue under the skin. Skin relaxation, atrophy, or edema of the connective tissue will all cause bags under the eyes, resulting in aging around the eyes and affecting people's external image [7]. Therefore, many beauty lovers pay attention to eye plastic surgery. Pouch surgery has substantial effect in the early stage, but there are many problems in the later stage, which is closely related to the formulation of the surgical plan, and the design of the surgical method needs to be determined according to the patient's own eye structure. To better understand the tissue structure of patients' eyes, we will use stereoscopic imaging technology to establish a three-dimensional (3D) model for evaluation in this study.

With the rapid development of artificial intelligence technology, people explore more and more deeply in the 3D world, and 3D technology today is more and more widely used. At present, 3D technology has been applied in the film industry [8], medical image processing [9], high-precision map [10], and intelligent detection [11]. Image feature extraction is relatively important in the process of 3D image establishment, and the convolutional neural network (CNN) algorithm is one of the common methods of 2D image recognition and information extraction at the present stage [12]. Studies showed that the application of CNN in deep learning is very mature and has been widely used in image feature extraction, classification, and other fields with excellent performance [13]. However, when there is a large amount of data to be processed, there will be a large error in the image processing of CNN. Therefore, someone proposed the three-channel CNN algorithm [14], that is, to process three sets of data at one time. However, there is a lack of research on the application of multichannel CNN model in ocular plastic surgery. Based on this, a multichannel CNN algorithm was established to analyze the three-dimensional model effect of reconstructing the eyelid tissue structure and used to assist the formulation of pouch surgery plan.

A three-dimensional model of eyelid tissue was established based on multichannel CNN algorithm to assist cosmetologists to develop a reasonable pouch surgery plan, and its auxiliary effect was evaluated by surgical results, aesthetic scores, and complications. It is aimed at obtaining methods to improve the effect of ocular plastic surgery and reduce the occurrence of postoperative adverse reactions in patients, so that patients are more satisfied.

## 2. Methods

### 2.1. The Research Object

64 patients who underwent blepharoplasty in XXX Hospital from January 2019 to March 2022 were studied. Among them, there were 26 male patients and 38 female patients, aged 28~60 years old, with mean age of 44.02 ± 6.27 years. 64 patients were randomly divided into observation group and control group. The patients in the observation group were evaluated by the ocular model based on multichannel CNN and the experience of the doctor to develop the surgical method, while the patients in the control group were evaluated only according to the experience of the doctor to develop the surgical method. The patients in a group were 32 cases. The auxiliary effect of multichannel CNN ocular model was evaluated by comparing the surgical effect and aesthetic score between the two groups. This study had been approved by the relevant medical ethics committee.

Inclusion criteria are as follows: (I) All patients had obvious pouch due to loose and bloated skin of lower eyelid. (II) All patients voluntarily underwent pouch surgery. (III) All patients had normal mental cognition and clear consciousness. (IV) All patients can cooperate with the normal operation. (V) All patients signed informed consent.

Exclusion criteria are as follows: (I) patients with visual impairment; (II) patients with dry eye; (III) patients with other heart diseases; (IV) patients with a history of ocular and periocular tissue surgery; (V) patients who took drugs affecting the experiment before surgery; and (VI) women in pregnancy or lactation.

### 2.2. Feature Extraction Based on CNN Algorithm

The feature extraction of CNN algorithm is carried out in the volume base, and feature extraction is carried out by convolution kernel. When the size of the image is large, it is necessary to slice the image, and the specific processing is as follows:

It is assumed that the size of the image is *A* × *B*, and the size of the image block is set to *a* × *b*; then, the number of image blocks *N* is

(1) $$\begin{array}{c} N=\frac{A\times B}{a\times b}. \end{array}$$

When *N* is not an integer, *N* is set to an integer by adding 0 to the image matrix. Then, the image is normalized to make the pixel values of image blocks more similar. The processing functions are as follows:

(2) $$\begin{array}{c} \hat{O}\left(x,y\right)=\frac{\left[\left(\text{O}-J\right)\left(x,y\right)\right]}{\left[\sigma \left(x,y\right)+z\right]}, \end{array}$$

(3) $$\begin{array}{c} J\left(x,y\right)=\frac{\left[\sum_{\Theta \in \left(k,l\right)}J\left(x+k,y+l\right)\right]}{\left[a^{\prime }\times b^{\prime }\right]}, \end{array}$$

(4) $$\begin{array}{c} \sigma \left(x,y\right)=\frac{\left[\sqrt{\sum_{\Theta \in \left(k,l\right)}\left(\left(x+k,y+l\right)-J\left(x,y\right)\right)}\right]}{\left[a^{\prime }\times b^{\prime }\right]}. \end{array}$$

In the above equations, (*x*, *y*) represents the pixel point of the image, *O* represents the pixel value at (*x*, *y*), *J* represents the mean value of the area block, *σ* represents the standard deviation of the area block, *z* represents any extremely small positive number (avoiding [*σ*(*x*, *y*) + *z*] = 0), Θ represents the local area of the calculation of *J* and *σ*, (*k*, *l*) represents the area block size, (*a*′, *b*′) represents the length and width of Θ, and $\hat{O}$ represents the pixel value of (*x*, *y*) after normalization. At this point, the mean value of the original image is 0, and the variance is 1.

After that, feature extraction is carried out on the image. The convolution layer of CNN is composed of many feature graphs, which are obtained by convolution kernel and upper input image through convolution operation. All the elements in the convolution kernel belong to the weight parameters, and the corresponding pixel value of the image needs to be multiplied and added by the convolution kernel and the pixel value of the input image and then obtained through the calculation of the activation function. Then, the matrix *W*_*v*_^*m*^ calculation expression corresponding to the first feature graph of the first layer is as follows:

(5) $$\begin{array}{c} W_{v}^{m}=f\left(\int_{i\in S_{v}}W_{v}^{m-1}\cdot l_{vi}^{m}+d_{v}^{m}\right). \end{array}$$

In the above equation, *f*(∫_*i*∈*S*_*v*__*W*_*v*_^*m*−1^ · *l*_*vi*_^*m*^ + *d*_*v*_^*m*^) represents the activation function, *S*_*v*_ represents the combination of feature graphs, *W*_*v*_^*m*−1^ represents the feature graph matrix of the upper layer, · represents the convolution processing, *l*_*vi*_^*m*^ represents the weight, and *d*_*v*_^*m*^ represents the bias.

### 2.3. CNN 3D Reconstruction Based on Multichannel

To reconstruct the image more carefully, the image is divided into blocks to the minimum range, which may lead to a large increase in image data. Therefore, it is necessary to suggest multichannel image processing to improve the processing efficiency and increase the similarity of image reconstruction.

The multichannel system is used to extract features from images. The multichannel CNN model is improved by the three-channel CNN model. The obtained feature images are combined to form a 3D image. Before reconstruction, it is necessary to estimate the probability of 3D images obtained from 2D feature images, mainly through threshold calculation. After input of image information, binarization threshold is obtained. Then, this value is applied to the probability model, and every pixel value is binarized to obtain the final 3D model. The specific process is as follows:

(6) $$\begin{array}{c} T=t\left(I\right), \end{array}$$

(7) $$\begin{array}{c} P=p\left(I\right), \end{array}$$

(8) $$\begin{array}{c} U=\int_{1,P_{i,j,k}\leq T}^{0,P_{i,j,k}>T}P_{i,j,k}\in P. \end{array}$$

In the above equations, *I* represents the characteristic image of input, *t* is the threshold calculation, *T* is the output threshold, *P* is the probability model, *p* is the basic network, *P*_*i*,*j*,*k*_ is the model probability of threshold calculation *P*, and (*i*, *j*, *k*) is the spatial coordinates of the model.

### 2.4. Evaluation Methods

The established three-dimensional model of the eye overlapped with the actual ocular structure of the patient to obtain similarity, and its reconstruction effect was evaluated by similarity and model reconstruction efficiency (the required time). The specific algorithm for similarity is as follows.

(9) $$\begin{array}{c} S=\left[\frac{V_{\text{coi}}}{V_{\text{total}}}\right]\times 100\%. \end{array}$$

In the above equation, similarity is represented by *S*, overlapping volume is represented by *V*_coi_, and total volume is represented by *V*_total_.

### 2.5. Observation Indicators

The effect of surgical treatment was evaluated by observing and comparing the changes of pouch degree, skin wrinkle depth of lower eyelid, lacrimal groove depth of eyelid, and skin gloss before and after treatment in the two groups. The assessment method of pouch degree is shown in Table 1, which was graded into mild 1 point, moderate 2 points, and severe 3 points. Fitzpatrick wrinkle grading method [15] was used to score skin wrinkles of lower eyelid, with scores ranging from 1 to 9. The lacrimal sulcus was evaluated by the degree of lacrimal sulcus filling [16], and the score was 0 ~ 10. Skin glossiness was detected by gloss meter glossiness meter [17], and the mean value was taken twice. The lower the Fitzpatrick wrinkle grading score, the higher the lacrimal filling degree and skin gloss, the better the treatment effect.

Aesthetic score [18] was used for aesthetic analysis of the eyes of patients in the two groups. Skin color, eye bag shape, eye contour, eye bag volume, and overall aesthetic feeling were analyzed. The score of each item was rated as 0, 1, or 2, and the total score was 10. The higher the score, the better the aesthetic effect.

The incidence of complications during treatment in the two groups was recorded and analyzed.

### 2.6. Statistical Methods

The SPSS 22.0 software was used for statistical analysis of the data in this study. The measurement data was expressed as mean ± standard deviation ($\overset{¯}{x}\pm s$), and the counting data was expressed as percentage (%). *T*-test was used to analyze eye related indicators and aesthetic scores, and *χ*^2^ test was used to analyze clinical efficacy and incidence of complications. 0.05 was taken as the test level, and *P* < 0.05 indicated statistically significant difference.

## 3. Results

### 3.1. Comparison of Reconstruction Effects

**Figure 1:**
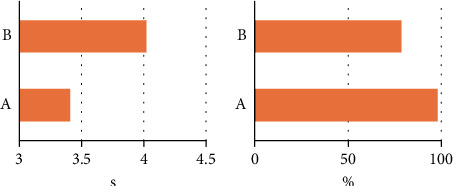
Reconstruction efficiency and similarity comparison. A, multichannel CNN algorithm; B, CNN algorithm. (a) Reconstruction efficiency; (b) similarity. Note: “^∗^” indicates that eye models based on multichannel CNN are more similar compared to traditional CNN models (*P* < 0.05).

**Figure 2:**
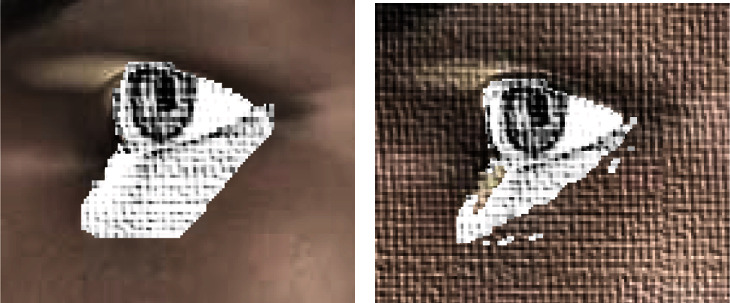
Overlap area correlation: (a) multichannel CNN algorithm; (b) CNN algorithm.

In this study, the multichannel CNN reconstruction algorithm was compared with the traditional CNN algorithm, and the reconstruction efficiency and similarity of the two stereo graphics were compared. The results showed that the reconstruction efficiency of the multichannel CNN algorithm was 3.41 s, which was basically consistent with the traditional CNN algorithm's reconstruction efficiency of 4.02 s, and there was no substantial statistical difference (*P* > 0.05). The similarity between the multichannel CNN model and the actual eye shape was 98.78%, which was considerably higher than that of the traditional CNN model (78.65%), and there was substantial statistical difference (*P* < 0.05) (Figures 1 and 2).

### 3.2. Comparison of General Clinical Data

**Figure 3:**
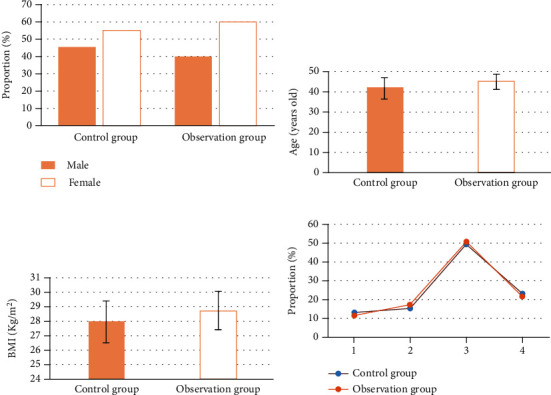
Comparison of general data: (a) gender; (b) age; (c) BMI; (d) educational attainment. 1, junior high school or below; 2, junior to senior high school; 3, high school ~ junior college/bachelor degree; 4, college/bachelor degree or above.

Through the statistics of the general data (gender, age, weight, education, etc.) of the two groups, in the control group, there were 14 male patients (45%) and 18 female patients (55%), with an average age of 42.21 ± 5.11 years, an average body mass index (BMI) of 28.02 ± 1.43 kg/m^2^, and 4 patients (12.5%) with below junior high school diploma, 5 patients (15%) with junior high school ~ senior high school diploma, 16 patients (50%) with senior high school ~ specialist/undergraduate diploma, and 7 patients (22.5%) with above specialist/undergraduate diploma. In the observation group, there were 13 male patients (40%) and 19 female patients (60%), with an average age of 45.45 ± 3.66 years, an average BMI of 28.77 ± 1.31 kg/m^2^, 3 patients (10%) with below junior high school diploma, 6 patients (17.5%) with junior high school ~ senior high school diploma, 17 patients (52.5%) with senior high school ~ specialist/undergraduate diploma, and 6 patients (20%) with above junior high school diploma. There were no statistically substantial differences in gender distribution, mean age, mean BMI, and educational background distribution between the two groups (*P* > 0.05) (Figure 3).

### 3.3. Comparison of Surgical Results

**Figure 4:**
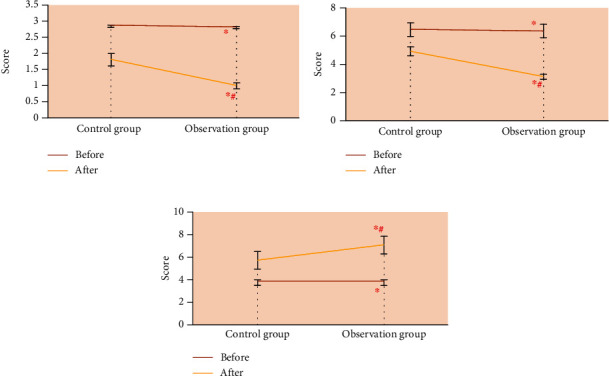
Comparison of scores of treatment indicators: (a) degree of eye bags; (b) skin wrinkles of lower eyelid; (c) skin gloss. Note: “^∗^” indicates that the pouch degree, lower eyelid skin wrinkles, and skin gloss score of patients in the two groups were significantly improved compared with those before operation (*P* < 0.05); “#” indicates that the pouch degree, lower eyelid skin wrinkles, and skin gloss score of patients in the observation group were better improved after treatment compared with those in the control group (*P* < 0.05).

Figure 4 shows the statistical results of pouch degree, skin wrinkles of lower eyelid, eyelid lacrimal sulcus, and skin gloss of the two groups before and after surgical treatment. Before treatment, the ocular scores in the control group were 2.89 ± 0.03 points, 6.39 ± 0.43 points, and 3.84 ± 0.21 points, respectively, and the scores in the observation group were 2.83 ± 0.04 points, 6.27 ± 0.42 points, and 3.76 ± 0.23 points, respectively. After comparison, there were no statistically substantial differences in pouch degree, lower eyelid skin wrinkle, eyelid lacrimal sulcus, and skin gloss score between the two groups before treatment (*P* > 0.05). After treatment, the results of ocular scores in the control group were 1.83 ± 0.18 points, 4.85 ± 0.23 points, and 5.76 ± 0.75 points, respectively, and the results of ocular scores in the observation group were 1.01 ± 0.08 points, 3.03 ± 0.14 points, and 7.12 ± 0.72 points, respectively. After comparison, the degree of pouch under the eyes, skin wrinkles of lower eyelid, eyelid lacrimal sulcus, and skin gloss scores of patients in the two groups were improved after treatment, and the improvement degree of various scores in the observation group was considerably better than that in the control group (*P* < 0.05).

### 3.4. Aesthetic Score Results

**Figure 5:**
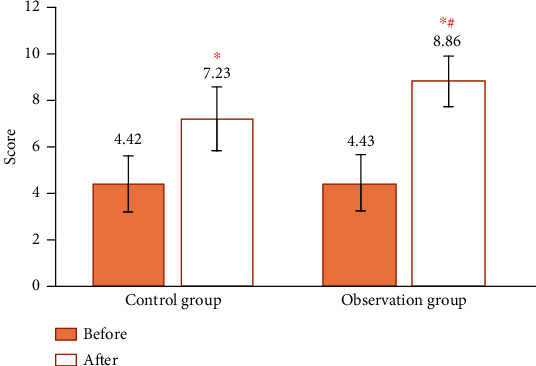
Comparison of aesthetic score between the two groups before and after treatment. Note: “^∗^” indicates that the aesthetic score of patients in the two groups was significantly improved compared with that before operation (*P* < 0.05); “#” indicates that the aesthetic score of patients in the observation group was better improved after treatment compared with that in the control group (*P* < 0.05).

Before treatment, the aesthetic score was 4.33 ± 1.17 points in the control group and 4.34 ± 1.15 points in the observation group. There was no significant difference in the aesthetic score between the two groups before treatment (*P* > 0.05). The aesthetic score was 7.09 ± 1.33 points in the control group and 8.68 ± 1.05 points in the observation group. After treatment, the ocular aesthetic score of the two groups was higher than that before treatment, and the aesthetic score of the observation group after treatment was higher than that of the control group (*P* < 0.05) (Figure 5).

### 3.5. Comparison of Complication Rates

**Figure 6:**
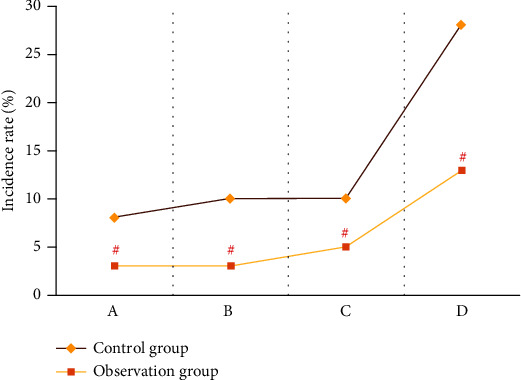
Comparison of complication rates: (a) lower eyelid ectropion; (b) lower eyelid retraction; (c) hematoma; (d) total complication rate. Note: “#” indicates that the incidence rate of complications in the observation group was lower than that in the control group (*P* < 0.05).

Through observation, the two groups of patients had postoperative complications such as lower eyelid eversion, lower eyelid retraction, and hematoma. The control group had 3 cases of lower eyelid eversion, 3 cases of lower eyelid retraction, and 3 cases of hematoma; the observation group had 1 case of lower eyelid eversion, 1 case of lower eyelid retraction, and 2 cases of hematoma; after analysis, the incidence rate of complications in the observation group (13%) was significantly lower than that in the control group (28%) (*P* < 0.05) (Figure 6).

## 4. Discussion

Due to the increasingly extensive pursuit of beauty today, it has greatly promoted the development of cosmetic and plastic surgery industry. Although successful cosmetic surgery has helped many people regain confidence in their appearance, there are still many cases of plastic surgery failure in clinical practice, which is more related to unreasonable surgical plans [18, 19]. To improve the effect of plastic surgery, CNN algorithm based on multichannel was adopted to establish 3D images of patients' eye structures to assist patients in making surgical plans.

The results showed that the efficiency of the multichannel CNN reconstruction algorithm (3.41 s) was basically consistent with that of the traditional CNN algorithm (4.02 s) (*P* > 0.05), while the reconstructed similarity (98.78%) was considerably higher than that of the traditional CNN algorithm (78.65%) (*P* < 0.05). According to the results, under the condition of the same reconstruction efficiency, the reconstruction algorithm based on multichannel CNN had a better effect, indicating that the multichannel system can improve the algorithm processing efficiency. This was consistent with the research results of Mughal and Ross [20]. Hu et al. [21] also mentioned in their study that multichannel CNN has good performance in processing multiple data parameters.

Abundant studies have shown that different pouch surgery methods have different therapeutic effects [22]. Therefore, it is very important to select reasonable and effective surgical methods to improve the aesthetic degree of patients' eyes. The degree of pouch, skin wrinkles of lower eyelid, skin gloss, and aesthetic score were compared to analyze the effect of surgical treatment, and the auxiliary effect of multichannel CNN eye model in pouch plastic surgery was evaluated. The results showed that, after treatment, the degree of pouch under the eyes, skin wrinkles of lower eyelid, skin gloss, and aesthetic score in the two groups were improved compared with before treatment, and the improvement degree of scores in observation group was considerably better than that in the control group (*P* < 0.05). It was suggested that the selection of surgical method and the formulation of surgical plan with the help of eye model can improve the effect of plastic surgery. Relevant studies have shown that to improve the symptoms of eye bags, canthus wrinkles, the rational release of orbital septal fat is relatively important [23, 24]. Different surgical methods have different therapeutic targets, such as orbital septal release surgery for the release of fat in the lower eyelid [25] and lateral canthus anchoring technology for the improvement of fullness and elasticity of the lower eyelid [26]. Therefore, the establishment of a model and the simulation of the surgical process are conducive to the improvement of the pertinence of the surgical plan. In addition, the incidence of complications was considerably lower in the observation group (13%) than in the control group (28%) (*P* < 0.05), indicating that reasonable and effective pouch surgery can alleviate the occurrence of complications after surgical treatment and improve the safety of surgery. This was consistent with a number of clinical research results, and the symptomatic degree of surgical method has a certain impact on the outcome of surgical treatment and the occurrence of postoperative complications [27–29]. Therefore, accurate examination and surgical method design is the pursuit of various clinical surgical treatment and is also very important to improve patient satisfaction, especially in the cosmetic and plastic surgery industry.

## 5. Conclusion

The effect of the 3D eye model based on the multichannel CNN algorithm in the formulation of reasonable pouch surgery plan by cosmetic surgeons was evaluated. The results showed that the eye model based on the multichannel CNN model could help improve the surgical repair and aesthetic effect of patients and could improve the occurrence of postoperative complications. However, there is no targeted comparative analysis of specific surgical methods in this study, which lacks certain comprehensiveness. Further treatment will be carried out later. Through this study, it is concluded that the development prospect of intelligent algorithm in the medical field is very considerable, worthy of further exploration and promotion of clinical research.

## Figures and Tables

**Table 1:** Evaluation criteria of pouch degree.

Degree	Manifestations	Score
Mild	The adipose bulge was located in the medial orbit without skin laxity	1
Moderate	Fat swelling was found in the medial and medial orbitals, with slight fine lines and basically no skin laxity	2
Severe	In, inside, and lateral adipose all bulge, and wrinkle and skin flabby phenomenon appears	3

## Data Availability

The datasets used in this paper are available from the corresponding author upon request.
